# Polyphenols as Potential Inhibitors of SARS-CoV-2 RNA Dependent RNA Polymerase (RdRp)

**DOI:** 10.3390/molecules26247438

**Published:** 2021-12-08

**Authors:** Yifei Wu, David Crich, Scott D. Pegan, Lei Lou, Madelyn C. Hansen, Carson Booth, Ellison Desrochers, Lauren Nicole Mullininx, Edward B. Starling, Kuan Y. Chang, Zhong-Ru Xie

**Affiliations:** 1 School of Electrical and Computer Engineering, College of Engineering, University of Georgia, Athens, GA 30602, USA; wuyifei@uga.edu (Y.W.); ll38965@uga.edu (L.L.); 2 Department of Pharmaceutical and Biomedical Sciences, College of Pharmacy, University of Georgia, Athens, GA 30602, USA; David.Crich@uga.edu; 3 Division of Biomedical Sciences, School of Medicine, University of California Riverside, Riverside, CA 92521, USA; scott.pegan@medsch.ucr.edu; 4 Franklin College of Arts and Sciences, University of Georgia, Athens, GA 30602, USA; Madelyn.Hansen@uga.edu (M.C.H.); CarsonBooth@uga.edu (C.B.); egd80862@uga.edu (E.D.); Lauren.Mullininx@uga.edu (L.N.M.); Edward.Starling@uga.edu (E.B.S.); 5 Department of Computer Science and Engineering, National Taiwan Ocean University, Keelung 202, Taiwan

**Keywords:** COVID-19, polyphenol, natural product, antiviral, molecular docking, MD simulation, remdesivir, drug discovery, virtual screening

## Abstract

An increasing number of studies have demonstrated the antiviral nature of polyphenols, and many polyphenols have been proposed to inhibit SARS-CoV or SARS-CoV-2. Our previous study revealed the inhibitory mechanisms of polyphenols against DNA polymerase α and HIV reverse transcriptase to show that polyphenols can block DNA elongation by competing with the incoming NTPs. Here we applied computational approaches to examine if some polyphenols can also inhibit RNA polymerase (RdRp) in SARS-CoV-2, and we identified some better candidates than remdesivir, the FDA-approved drug against RdRp, in terms of estimated binding affinities. The proposed compounds will be further examined to develop new treatments for COVID-19.

## 1. Introduction

Polyphenols, commonly found in plants, fruits, and tea, are antioxidants with many health benefits [1,2]. Previous studies have shown polyphenols hold antiviral properties against various viruses [3,4,5,6], especially those targeting the respiratory tract [7,8,9,10]. Moreover, many polyphenols were found to inhibit SARS-CoV or its target proteins [11,12,13,14,15,16,17,18,19,20,21,22,23,24,25,26]. The anti-inflammatory properties of polyphenols are strong enough that they have been suggested as a supplement for obese and elderly COVID-19 patients [1]. Considerations like these have prompted researchers to investigate polyphenols’ ability to inhibit SARS-CoV-2 proteins. In an in silico analysis performed by Singh et al., TF3, TF2b, TF1, TF2a, and hesperidin all had better binding scores compared to remdesivir [27]. Though not better than remdesivir, gallic acid had a large binding energy when docked to RdRp in a study of plant polyphenols by Nourhan et al., showing strong inhibitory potential [28]. Despite the promising results, these studies sampled a very small variety of polyphenols, mainly tea extracts. Therefore, it is necessary to conduct large-scale virtual screening for a larger number of polyphenols.

Since it was first identified in late 2019, SARS-CoV-2 has devastated the world, infecting more than 200 million people and killing more than 4.7 million [29]. In December 2020, the FDA approved the Pfizer-BioNTech vaccine for emergency use, resulting in more than 100 million vaccinations in the U.S. alone [30]. Despite the success of the vaccines, COVID-19 cases continue to increase, making the discovery of safe antiviral drugs a significant concern. 

SARS-CoV-2 is an enveloped, positive-sense RNA virus closely related to SARS-CoV, the cause of the SARS pandemic in 2002–2003 [31,32]. The virus’ genome consists of 14 ORFs, with ORF 1a and 1b being the most important for RNA replication [33,34]. After entry into a cell, ORF 1a and 1b are translated into two polyproteins that are further broken down into 16 non-structural proteins (nsps) [33]. These proteins assemble to form a replication complex that transcribes and replicates the virus’ RNA [35]. This RNA is ultimately packaged to make new SARS-CoV-2 viruses. 

As the enzyme complex responsible for generating RNA, RNA-dependent RNA polymerase (RdRp) is central to viral replication [32]. The main protein in the complex is nsp12 bound by two accessory subunits, nsp 7 and nsp8 [36]. Given that human cells do not use RdRp and that its role in the viral life cycle is critical, RdRp is a popular drug target [37,38]. Inhibitors of RdRp fall into two categories: nucleoside inhibitors (NI) and non-nucleoside inhibitors (NNI). After being incorporated, nucleoside inhibitors block new nucleotides from entering the RNA chain, so synthesis stops [36]. In an in vitro study using a newly developed CoV-RdRp-Gluc reporter assay, remdesivir and molupiravir, both NIs, inhibited RdRp with EC_50_ values of 11.11 and 0.22 µM, respectively [32]. NNIs inhibit RdRp by changing its shape after binding to allosteric sites on the protein [36]. In a molecular docking study by Zijing et al., tegobuvir, an NNI drug in development for hepatitis C, bonded to the nsp12-7 and nsp12-8 sections of RdRp with docking scores ranging from −7.8 to −8.4 kcal/mol, showing an inhibitory effect [39]. Compared to NIs, which are vulnerable to exonuclease (nsp14) cleavage and competition with nucleotides present in the cell, NNIs have the potential to be highly potent drugs against SARS-CoV-2 [32,36].

Thus, to find potential therapeutic agents against COVID-19, this study screened 480 polyphenols to bind to SARS-CoV-2 RdRp. The structures of the 480 polyphenols were obtained from the Phenol-Explorer 3.6 database [40,41,42], and molecular docking was conducted using Maestro. Molecular mechanics with generalized Born and surface area solvation (MM-GBSA) scores were collected to quantify the affinity of the molecules for the proteins, and then absorption, distribution, metabolism, and excretion (ADME) and drug-likeness properties were analyzed for further screening. This study identifies three polyphenols with extremely low binding affinities to the SARS-CoV-2 RdRp as potential natural products for COVID-19 treatment.

## 2. Results

### 2.1. Docking Analysis of Polyphenols against SARS-CoV-2 RdRp

To develop effective inhibitors from polyphenols against SARS-CoV-2 RdRp, 480 polyphenols were docked onto SARS-CoV-2 RdRp (PDB ID: 7BV2). Based on docking poses, the binding energies were calculated using Prime MM-GBSA in Maestro. As a result, the top three protein–ligand complexes, namely RdRp–cyanidin 3-*O*-rutinoside (−107.68 kcal/mol), RdRp–petunidin 3,5-*O*-diglucoside (−99.18 kcal/mol), and RdRp–delphinidin 3-*O*-rutinoside (−90.70 kcal/mol), have better estimated binding energies (Table 1). These three polyphenols belong to the group of anthocyanins [43,44,45]. Meanwhile, we selected four compounds—remdesivir-TP (−55.00 kcal/mol), theaflavin 3,3′-digallate (TF3) (−77.89 kcal/mol), swertiapuniside (−39.42 kcal/mol), and ATP (−57.83 kcal/mol)—as a control group. Here, remdesivir-TP is the best drug candidate, which was identified in our previous study [46]. TF3 and swertiapuniside were proposed as the top-ranked inhibitors of RdRp in the studies from Singh et al. [27] and Koulgi et al. [47], respectively. From Table 1, we find that the top three polyphenols show better binding energies than remdesivir-TP, TF3, and swertiapuniside. This result indicates that the top three polyphenols possess the potential to inhibit RdRp. Furthermore, the binding energies of the top three polyphenols are better than that of ATP, which also suggests that the top three polyphenols might exert strong competitiveness at the ATP binding site.

**Figure 1:**
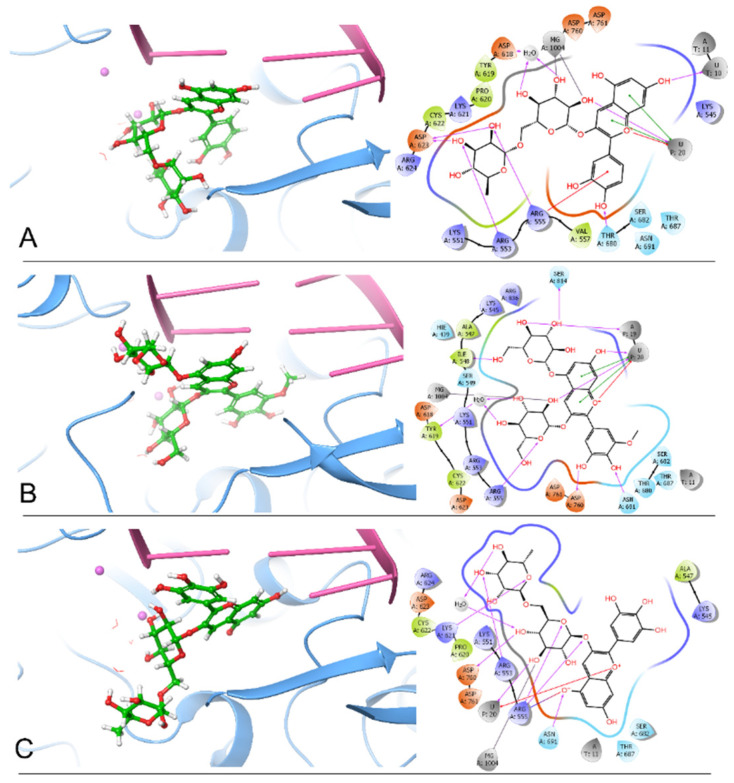
The docking poses and 2D ligand–protein interaction diagrams of 7BV2 and the top three ligands: (**A**), Cyanidin 3-*O*-rutinoside; (**B**), Petunidin 3,5-*O*-diglucoside; (**C**), Delphinidin 3-*O*-rutinoside. The pink spheres represent Mg^2+^ ions. The purple arrows indicate the hydrogen bonds; the green line represents π-π stacking; the red line represents π–cation interaction; the blue-red line represents the salt bridge; the gray line represents metal coordination.

By comparing the 2D ligand–protein interactions of the top three polyphenols bound to RdRp (Figure 1), we find that they all interact with U20, the terminal nucleotide that interacts with incoming ATP. Cyanidin 3-*O*-rutinoside interacts with U20 by forming one hydrogen bond, two π-π stackings, and one π–cation interaction. By forming two hydrogen bonds, two π-π stackings, and one π–cation interaction, petunidin 3,5-*O*-diglucoside interacts with U20. Meanwhile, delphinidin 3-*O*-rutinoside interacts with U20 by forming one hydrogen bond and one π–cation interaction. These interactions between the top three polyphenols and U20 suggest that these polyphenols might strongly bind to RNA and compete with incoming ATP for the binding site so that viral RNA elongation is blocked. 

Additionally, we summarized the number of hydrogen bonds between the top three polyphenols and the essential residues in Table 2. From Table 2, we find that these three polyphenols all interact with Arg555 by forming hydrogen bonds: one hydrogen bond for cyanidin 3-*O*-rutinoside, one hydrogen bond for petunidin 3,5-*O*-diglucoside, and two hydrogen bonds for delphinidin 3-*O*-rutinoside. Moreover, cyanidin 3-*O*-rutinoside interacts with Arg555 by forming a π–cation interaction, and delphinidin 3-*O*-rutinoside interacts with Agr555 through a salt bridge (Figure 1A,C). This result suggests that Arg555 is a critical residue in the binding pocket. Furthermore, cyanidin 3-*O*-rutinoside and delphinidin 3-*O*-rutinoside both interact with Arg553 by forming one hydrogen bond each. In forming one hydrogen bond each, petunidin 3,5-*O*-diglucoside and delphinidin 3-*O*-rutinoside both interact with Asn691 and Asp760. Notably, the ligand delphinidin 3-*O*-rutinoside has a phenoxide ion interacting with Arg555 and Asn691 (Figure 1C). Accordingly, we propose that cyanidin 3-*O*-rutinoside, petunidin 3,5-*O*-diglucoside, and delphinidin 3-*O*-rutinoside are the three best inhibitor candidates of RdRp among all 480 polyphenols based on these docking results. 

### 2.2. Physicochemical Properties Prediction

To explore the physicochemical properties of the top three polyphenols, we predicted the ADME and drug-likeness properties using Qikprop in Maestro. The results are shown in Table 3. Meanwhile, the physicochemical properties of three control compounds are also predicted and listed in Table 3. First, the molecular weight of the top three polyphenols is within the recommended range of 130.0 to 725.0, whereas the control compound TF3 falls out of the recommended range. QPlogS is another important property to predict aqueous solubility, and its recommended range is from −6.5 to 0.5. All the compounds in Table 3 are in that range. In addition, the Lipinski’s rule of five (RO5) and the Jorgensen’s rule of three (RO3) are essential descriptors for evaluating drug-likeness. The top three polyphenols all pass the RO5 and RO3, which further suggests the drug-likeness of these polyphenols. In summary, Qikprop predictions indicate that cyanidin 3-*O*-rutinoside, petunidin 3,5-*O*-diglucoside, and delphinidin 3-*O*-rutinoside can be considered as viable drug candidates worthy of further research.

### 2.3. Molecular Dynamics (MD) Simulation Analysis

**Figure 2:**
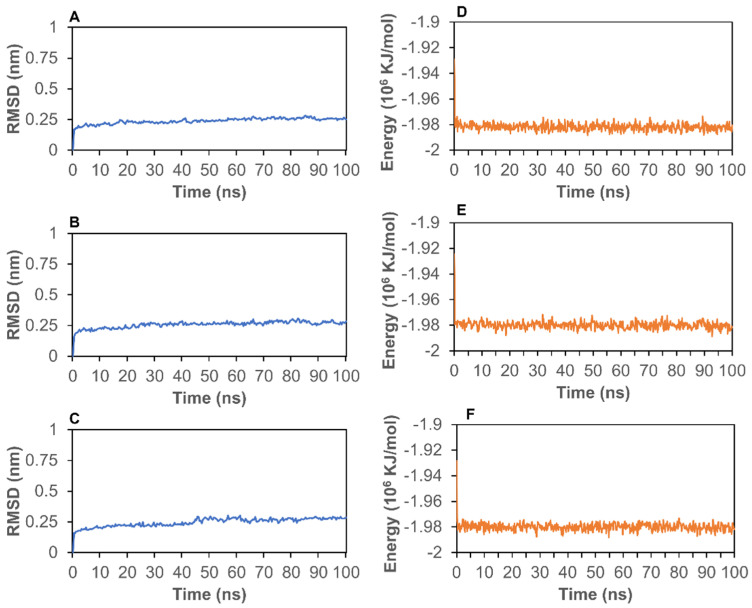
The RMSD and energy of protein–ligand complexes. (**A**), the RMSD of 7BV2–cyanidin 3-*O*-rutinoside; (**B**), the RMSD of 7BV2–petunidin 3,5-*O*-diglucoside; (**C**), the RMSD of 7BV2–delphinidin 3-*O*-rutinoside; (**D**), the energy of 7BV2–cyanidin 3-*O*-rutinoside; (**E**), the energy of 7BV2–petunidin 3,5-*O*-diglucoside; (**F**), the energy of 7BV2–delphinidin 3-*O*-rutinoside.

To further analyze the stability of the complexes, we conducted MD simulations to calculate RMSD and energy for the top three protein–ligand complexes. First, RMSD can be used to assess the stability of a protein–ligand complex. As shown in Figure 2A–C, the RMSD for the 7BV2–ligand complexes stabilized at around 0.25 nm after 5 ns, which suggests that the protein–ligand complexes are stable during the simulation process. Moreover, the total energies of these three complexes are shown in Figure 2D–F. The energies of 7BV2–ligand complexes stabilized at around −1.98 × 10^6^ kJ/mol after 2 ns, further revealing the good stabilities of these three systems. 

**Figure 3:**
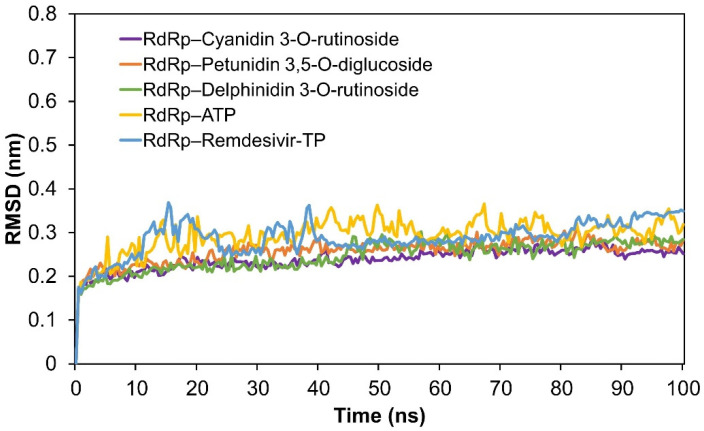
The comparison of the RMSD of protein–ligand complexes. The purple, red, green, yellow, and blue lines represent the RMSD of complexes of RdRps binding to cyanidin 3-*O*-rutinoside, petunidin 3,5-*O*-diglucoside, delphinidin 3-*O*-rutinoside, ATP and remdesivir, respectively.

To compare the stability of RdRp with the three best polyphenols and the control group, we conducted MD simulations on the control complexes: RdRp–remdesivir-TP and RdRp–ATP. The RMSD values were then calculated and shown in Figure 3. As a result, the RMSD values of RdRp–remdesivir-TP and RdRp–ATP were higher than those of the complexes of RdRp with the three best ligands, which suggests that these three top-scored polyphenols, bound to RdRp, show better stability. Figure S1 shows that the RMSD values of 7BV2 before and after docking all stabilize at around 0.25 nm from 5 ns to 100 ns. Interestingly, the RMSD value of 7BV2 before ligand docking is slightly higher than the others, which indicates that the protein structure of RdRp becomes more stable after ligand–protein docking. Therefore, we conclude that the top three polyphenols bound to RdRp promote the stability of the protein structure. After MD simulations, we created three 2D interaction diagrams between RdRp and the three top-scored polyphenols (Figure S2). Compared to the docking results, the number of interactions (hydrogen bonds and π–π stacking) was reduced (Figure 1 vs. Figure S2A–C) because the fluctuation of the structures is the nature of MD simulations. However, we also found that the most significant interactions between the ligands and the protein remained stable (Figure S2). For example, the hydrogen bond, π–cation, and π–π interactions between the top-scored polyphenols and Arg555 and U20, the terminal nucleotide, remained stable during the MD simulations. As the control, the complex of RdRp and remdesivir-TP had fewer interactions after the MD simulation, while remdesivir-TP still interacted with U20 but not with Arg555 (Figure S3).

## 3. Discussion

Polyphenols possess a wide range of health benefits and biological activities, including antioxidant [50], antitumoral [51], anti-inflammatory [52], and antiviral properties [53], the latter of which suggests that polyphenols may be useful in the battle against multiple viruses, even SARS-CoV-2 [27,54,55,56,57,58,59,60,61,62]. In fact, numerous studies have shown that polyphenols exhibit antiviral effects by inhibiting DNA/RNA synthesis [63,64,65,66,67]. Our previous study also demonstrated that miquelianin, a flavonol glucuronide, can compete for the binding site of dATP on HIV-1 reverse transcriptase, inhibiting the viral DNA synthesis by interacting with the last nucleotide of the RNA chain and the binding residues of DNA polymerase [68]. Competing for the binding site of the incoming dNTP on the 3′ end of the elongating chain of DNA or RNA on the DNA/RNA polymerases is a significant mechanism through which polyphenols or other artificial compounds such as remdesivir exert their inhibitory effects. Therefore, to virtually screen potential inhibitors against DNA/RNA polymerases, the DNA or RNA molecule has to be considered and located precisely in the docking box. However, compounds were docked onto the empty active site of RdRp of SARS-CoV-2 without the RNA chain involved in many previous studies. This should be a reason why many drug candidates identified in in silico experiments are false positives.

**Figure 4:**
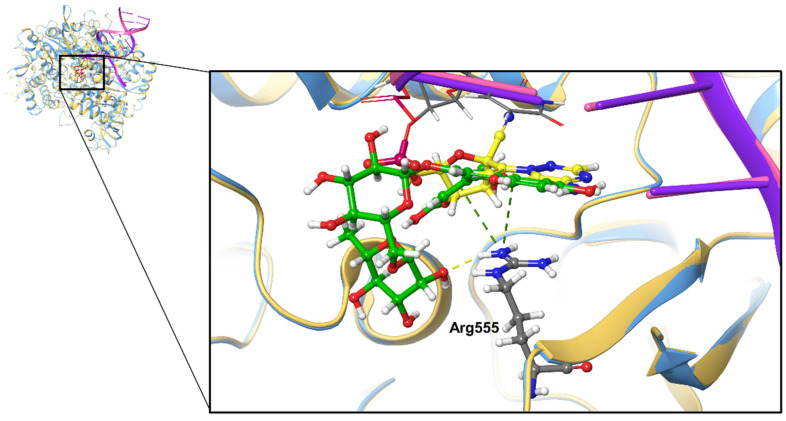
Superposition of cyanidin 3-*O*-rutinoside docked onto SARS-CoV-2 RdRp (7BV2) and cryo-EM structure of SARS-CoV-2 RdRp with remdesivir-MP (7BV2). The yellow-colored compound represents remdesivir-MP on 7BV2 (yellow-colored); the green-colored compound represents cyanidin 3-*O*-rutinoside docked on 7BV2 (blue-colored). The yellow dash line represents a hydrogen bond, and the green dash line represents a π–cation interaction.

In this study, we found that the binding affinities of the top-ranked polyphenols all showed much better results than remdesivir in terms of binding affinity. To further discuss the docking pose of the best drug candidate, we overlapped the docking pose of 7BV2–cyanidin 3-*O*-rutinoside with the original structure of 7BV2 (Figure 4). We found that cyanidin 3-*O*-rutinoside occupies the position of the original ligand remdesivir-MP, but that it also interacts with Arg555 and the last nucleotide of the RNA chain in addition to the interactions between RdRp and the original ligand remdesivir-MP. Cyanidin 3-*O*-rutinoside interacts with Arg555 by forming one hydrogen bond and one π–cation interaction; however, remdesivir-MP interacts with Arg555 only through one π–cation interaction. Cyanidin 3-*O*-rutinoside interacts with U20, the last nucleotide, through a π–π interaction as well. These findings indicate that cyanidin 3-*O*-rutinoside, the polyphenol with the best scores, should compete for the binding site to block viral RNA synthesis. 

## 4. Materials and Methods

### 4.1. Ligand Preparation

The structures of 480 tested polyphenols were retrieved from Phenol-Explorer 3.6 (http://phenol-explorer.eu/) (accessed on 1 December 2020). All the tested compounds were prepared using Ligprep in Maestro 12.4 (Schrödinger). The process for Ligprep includes adding hydrogens, computing correct partial charges, and generating possible conformations. The force field is OPLS3e by default [69].

### 4.2. Protein Preparation

The protein structures of RNA-dependent RNA polymerase (PDB ID: 7BV2) from RCSB’s Protein Data Bank (https://www.rcsb.org/) were prepared for use by Maestro in three steps: preprocessing, optimization, and minimization [70,71]. The OPLS3e force field was applied in both the optimization and the minimization steps [69]. 

### 4.3. Ligand–Protein Docking

To estimate the interactions between target proteins and polyphenols, we conducted ligand–protein docking by using the Ligand Docking panel in Maestro. Before running docking jobs, a receptor grid box was generated based on the existing ligand remdesivir in the protein structure. The size of the receptor grid box was set as default (20 Å). Ligand–protein docking was performed in extra-precision (XP) mode.

### 4.4. MM-GBSA Calculation

To predict the binding energies of polyphenols bound to RdRp, we performed Prime MM-GBSA (molecular mechanics generalized Born surface area) in Maestro. In the MM-GBSA panel, the pose viewer files of the docked complex were uploaded into the MM-GBSA panel. The force field was OPLS3e [69].

### 4.5. ADME and Drug-Likeness Properties Prediction

Qikprop module in Maestro was applied to predict the absorption, distribution, metabolism, and excretion (ADME) and drug-likeness properties for further screening [72]. For Qikprop, the top-ranked polyphenols were prepared by using Ligprep. Finally, descriptors such as RuleOfFive (RO5) and RuleOfThree (RO3) were applied to analyze the candidates.

### 4.6. Molecular Dynamics Simulation

To further investigate the dynamic interactions between RdRp and the top three polyphenols, we conducted molecular dynamics (MD) simulations by using GROMACS version 2018.1 and CHARMM36 force field [73]. The starting coordinates of the protein–ligand complex were obtained from a ligand–protein docking study. Then, we used CHARMM-GUI to build the MD simulation solution box, a cubic box with a length of 125 Å, which was then filled with water [74,75,76]. Next, the minimized structures were equilibrated using an NVT ensemble (constant Number of particles, Volume, and Temperature) and NPT ensemble (the Number of particles, Pressure, and Temperature). The target equilibration temperature was 300 K. Finally, MD simulations were performed for 100 ns. After the MD simulations, we calculated the root-mean-square deviation (RMSD) and the potential energies.

## 5. Conclusions

In summary, this study identified three polyphenols out of 480 as the best drug candidates for COVID-19 treatment. They all showed better estimated binding affinities than control compounds such as remdesivir. This can offer inspiration for new drug development.

## Figures and Tables

**Table 1:** The estimated binding energies of the top three polyphenols and the control compounds.

Compound	Binding Energy (kcal/mol)
Cyanidin 3-*O*-rutinoside	−107.68
Petunidin 3,5-*O*-diglucoside	−99.18
Delphinidin 3-*O*-rutinoside	−90.70
Remdesivir-TP ^1^	−55.00
TF3 ^2^	−77.89
Swertiapuniside ^3^	−39.42
ATP ^4^	−57.83

^1^ The original ligand in 7BV2 and the best-scored potential drug identified by our previous study [46]. ^2^ The best-scored potential drug identified by a previous study [27]. ^3^ The best-scored potential drug identified by a previous study [47]. ^4^ The substrate of RNA synthesis.

**Table 2:** The number of hydrogen bonds formed between the top three polyphenols and essential residues of SARS-CoV-2 RdRp.

	Cyanidin 3-*O*-rutinoside	Petunidin 3,5-*O*-diglucoside	Delphinidin 3-*O*-rutinoside
Ile548		1	
Arg553	1		1
Arg555	1	1	2
Lys621			2
Asp623	2		
Thr680	1		
Asn691		1	1
Asp760		1	1
Ser814		1	

**Table 3:** Selected Qikprop descriptors of the top three polyphenols and the control compounds.

Compound	mol_MW ^1^	QPlogS ^2^	RO5 ^3^	RO3 ^4^
Cyanidin 3-*O*-rutinoside	596.541	−2.112	3	2
Petunidin 3,5-*O*-diglucoside	642.566	−1.187	3	2
Delphinidin 3-*O*-rutinoside	612.540	−2.663	3	2
Remdesivir-TP	531.205	−1.742	3	1
TF3	868.714	−4.852	3	2
Swertiapuniside	531.205	−1.742	3	1

^1^ mol_MW represents molecular weight of the molecule. The recommended range is 130.0–725.0. ^2^ QPlogS is the predicted aqueous solubility. The recommended range is −6.5~0.5. ^3^ RO5: number of violations of Lipinski’s rule of five [48]. The recommended range: maximum is 4. ^4^ RO3: Number of violations of Jorgensen’s rule of three [49]. The recommended range: maximum is 3.

## Data Availability

The data presented in this study are available on request from the corresponding author.

## Supplementary Materials

The following are available online, Figure S1: The RMSD of 7BV2 before and after ligand-protein docking. Figure S2: 2D ligand-protein interaction diagrams of RdRp and the top three ligands after MD simulations: A, Cyanidin 3-*O*-rutinoside; B, Petunidin 3,5-*O*-diglucoside; C, Delphinidin 3-*O*-rutinoside. Figure S3: 2D ligand-protein interaction diagrams of RdRp-remdesivir-TP before (A) and after (B) MD simulation.
