# Supplementary material for: Polyphenols as Potential Inhibitors of SARS-CoV-2 RNA Dependent RNA Polymerase (RdRp)
Source: Molecules. 2021 Dec 8;26(24):7438. doi: 10.3390/molecules26247438 (PMC8706955; doi:10.3390/molecules26247438)
Supplement: Supplementary file 1 [file molecules-26-07438-s001.zip › molecules-1438034-supplementary.pdf]

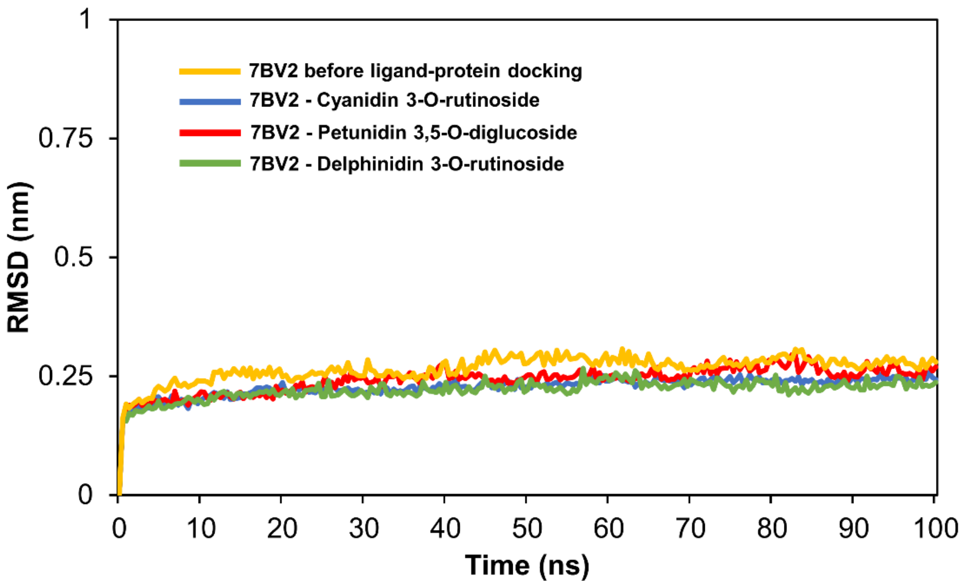

Figure S1 The RMSD of 7BV2 before and after ligand-protein docking. The yellow line represents the RMSD of 7BV2 before ligand-protein docking; the blue line represents the RMSD of 7BV2 after binding of cyanidin 3-O-rutinoside; the red line represents the RMSD of 7BV2 after binding of petunidin 3,5-O-diglucoside; the green line represents the RMSD of 7BV2 after binding of delphinidin 3-O-rutinoside.

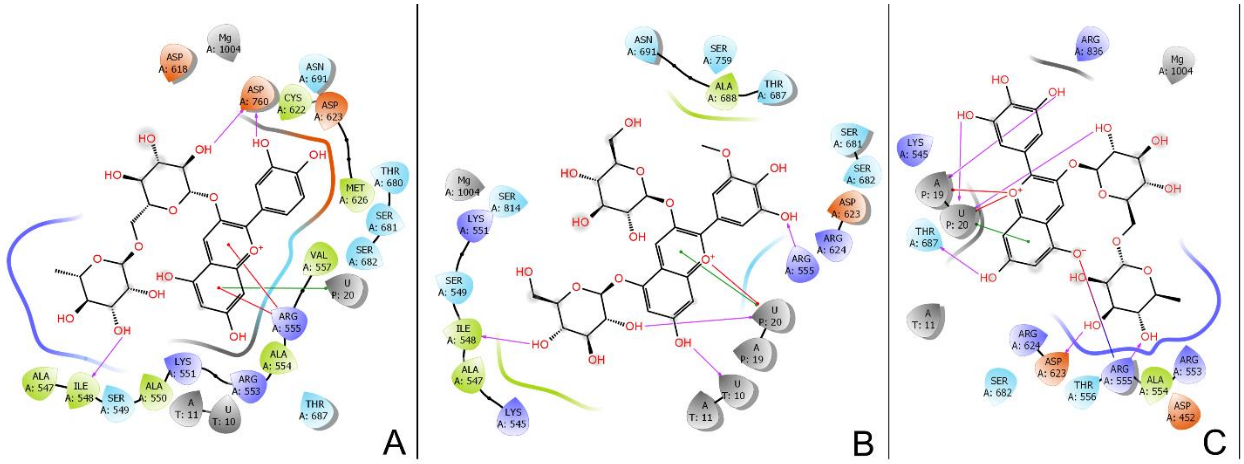

Figure S2 2D ligand-protein interaction diagrams of RdRp and the top three ligands after MD simulations: A, Cyanidin 3-O-rutinoside; B, Petunidin 3,5-O-diglucoside; C, Delphinidin 3-O-rutinoside. The pink spheres represent Mg^{2+} ions. The purple arrows indicate the hydrogen bonds; the green line represents π-π stacking; the red line represents π -cation interaction; the blue-red line represents the salt bridge.

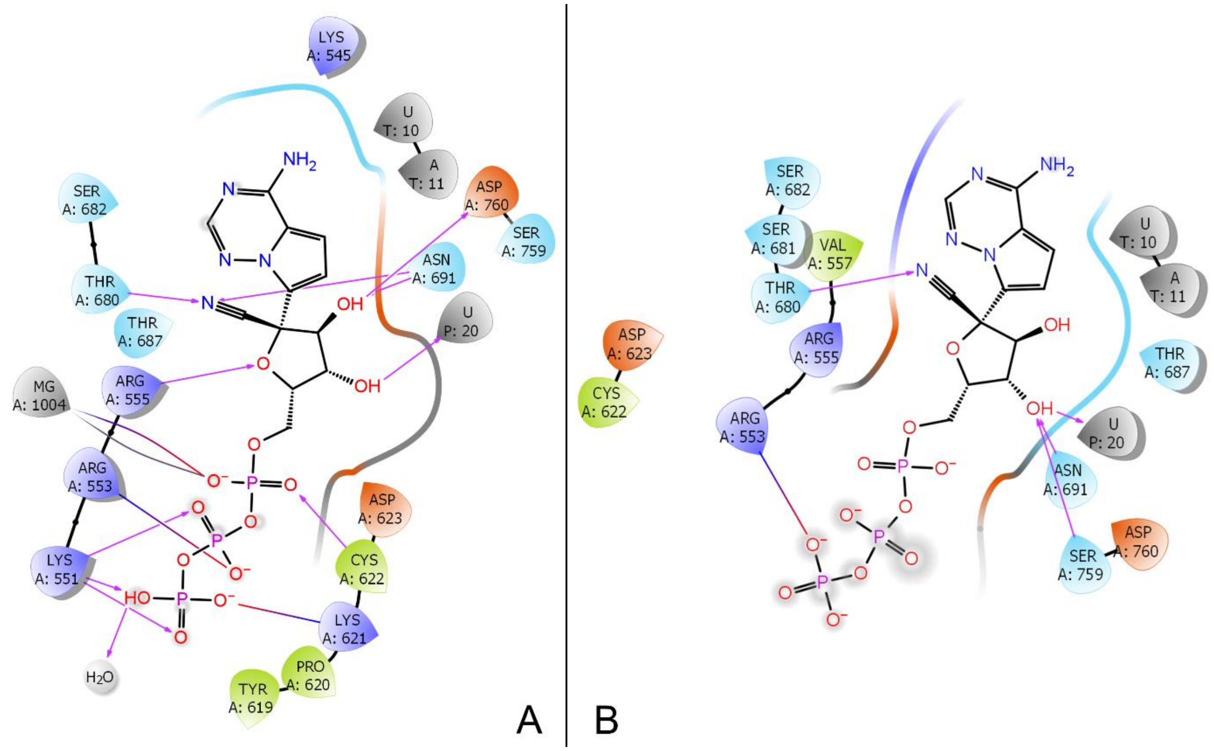

Figure S3 2D ligand-protein interaction diagrams of RdRp-remdesivir-TP before (A) and after (B) MD simulation. The purple arrows indicate the hydrogen bonds; the blue-red line represents the salt bridge; the gray line represents metal coordination.
